# Evaluation of Antioxidant Activity of Spice-Derived Phytochemicals Using Zebrafish

**DOI:** 10.3390/ijms21031109

**Published:** 2020-02-07

**Authors:** Yuka Endo, Kyoji Muraki, Yuji Fuse, Makoto Kobayashi

**Affiliations:** Department of Molecular and Developmental Biology, Faculty of Medicine, University of Tsukuba, Tsukuba, Ibaraki 305-8575, Japan; s1821258@s.tsukuba.ac.jp (Y.E.); s1921309@s.tsukuba.ac.jp (K.M.); fuseyk@gmail.com (Y.F.)

**Keywords:** antioxidant activity, food ingredient, H_2_O_2_, 6-MSITC, NaAsO_2_, Nrf2, oxidative stress, dietary phytochemical, spice, zebrafish

## Abstract

Various dietary phytochemicals seem to display antioxidant activity through the NF-E2-related factor 2 (Nrf2) pathway. However, few studies have demonstrated its antioxidant effect and Nrf2 dependency at the animal level. We constructed a zebrafish-based assay system to analyze the in vivo antioxidant activity of phytochemicals and examined the activity of 10 phytochemicals derived from spices, using this system as a pilot study. Hydrogen peroxide and arsenite were used as oxidative stressors, and Nrf2 dependency was genetically analyzed using an Nrf2-mutant zebrafish line. The activities of curcumin, diallyl trisulfide and quercetin were involved in the reduction of hydrogen peroxide toxicity, while those of cinnamaldehyde, isoeugenol and 6-(methylsulfinyl)hexyl isothiocyanate were involved in the reduction of arsenite toxicity. The antioxidant activities of these phytochemicals were all Nrf2 dependent, with the exception of cinnamaldehyde, which showed strong antioxidant effects even in Nrf2-mutant zebrafish. In summary, we succeeded in constructing an assay system to evaluate the in vivo antioxidant activity of various phytochemicals using zebrafish larvae. Using this system, we found that each spice-derived phytochemical has its own specific property and mechanism of antioxidant action.

## 1. Introduction

Oxidative stress has various harmful effects in animal cells and leads to serious diseases and accelerates the aging process [1,2]. Thus, reducing oxidative stress in the human body is expected to prevent the progression of diseases and aging [3]. One effective and economical strategy for reducing oxidative stress in the body is to consume antioxidant phytochemicals that are included in various foods, such as curcumin, quercetin and sulforaphane [4,5]. These dietary phytochemicals are considered to activate and/or induce several antioxidant proteins in host cells, mainly via the NF-E2-related factor 2 (Nrf2) pathway [6,7]. The Nrf2 pathway is an evolutionarily conserved cellular defense system against oxidative stress that induces the expression of antioxidant proteins at the transcriptional level and which indirectly eliminates oxidative stress [8,9]. Nrf2-knockout mice are viable and fertile but susceptible to various kinds of lifestyle-related diseases, the onset and progression of which seem to be facilitated by endogenous oxidative stress [10]. Interestingly, a number of phytochemicals that have been reported to activate the Nrf2 pathway are expected to prevent lifestyle-related diseases and extend healthy life expectancy in humans [11]. Among these Nrf2-activating phytochemicals, an isothiocyanate, sulforaphane, which is found in broccoli and other cruciferous vegetables, is the most extensively studied [12]. Sulforaphane is a promising agent that is under preclinical evaluation in many models of disease prevention [13].

The antioxidant activity of sulforaphane has been clearly shown to be Nrf2 dependent by the analyses of knockout animals [14,15]. With regard to phytochemicals other than sulforaphane, however, they have been mainly analyzed only by the induced activity of Nrf2 target genes or reporters, and the possible involvement of other biological pathways in these antioxidant activities has remained unexplored. Genetic analyses have not been performed primarily for economic reasons and the ethical difficulties associated with the use of Nrf2-knockout mice in the evaluation of the antioxidant activities of phytochemicals. It is also difficult to prepare high-quality Nrf2-knockout cells using appropriate cell lines. In contrast, zebrafish might be useful for a comprehensive phytochemical analysis, since it is easy to prepare thousands of embryos/larvae at once and to perform drug treatments using them [16]. We have studied the Nrf2 pathway using zebrafish for two decades and found that the functions and regulatory mechanism of the Nrf2 pathway are highly conserved among vertebrates [17,18,19,20,21,22,23,24,25,26]. We also established a mutant line of zebrafish Nrf2 (*nfe2l2a^fh318^*) and demonstrated that the antioxidant activity of zebrafish larvae was increased by the treatment of sulforaphane and other Nrf2-activating compounds in an Nrf2-dependent manner [15,27,28].

In this study, we evaluated the antioxidant activity of 10 spice-derived phytochemicals (capsaicin, carnosic acid, cinnamaldehyde, curcumin, diallyl trisulfide, eugenol, 6-gingerol, isoeugenol, 6-(methylsulfinyl)hexyl isothiocyanate (6-MSITC) and quercetin) and analyzed their Nrf2 dependency using wild-type and Nrf2-mutant zebrafish. Two types of oxidative stressors (hydrogen peroxide (H_2_O_2_)) and sodium arsenite (NaAsO_2_)) were used in the analyses. As a result, each phytochemical showed different antioxidant activity, and, interestingly, some showed stressor-specific activity. In addition, we found that there were both Nrf2-dependent and Nrf2-independent activities.

## 2. Results

### 2.1. Establishment of the Zebrafish-Based Assay System for Evaluating the Antioxidant Activities of Phytochemicals

**Figure 1:**
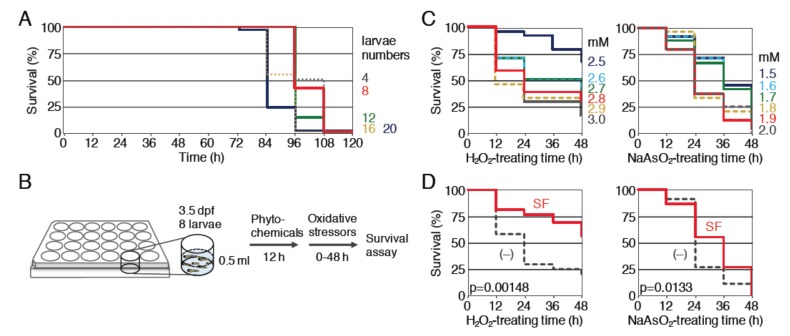
Survival assays of zebrafish larvae against oxidative stressors using 24-well plates. (**A**) Determination of the numbers of larvae. The indicated numbers of larvae (4 dpf) were placed into each well and survival rates were evaluated for 120 h without exchanging the medium. Larvae numbers: 4 (gray, dotted), 8 (red), 12 (green), 16 (yellow, dotted), 20 (purple). (**B**) A schematic diagram of the survival assay. Eight larvae (3.5 dpf) were placed into a single well of a 24-well plate, pretreated with phytochemicals for 12 h, then treated with oxidative stressors for 48 h, and the survival rates of the treated larvae were measured every 12 h. (**C**) Determination of the optimal concentration of oxidative stressors for the survival analysis. Larvae (4 dpf) were exposed to H_2_O_2_ at concentrations of 2.5 to 3.0 mM (**left panel**; 2.5 (dark blue), 2.6 (light blue, dotted), 2.7 (green), 2.8 (red), 2.9 (yellow, dotted), 3.0 mM (gray)) or NaAsO_2_ at concentrations of 1.5 to 2.0 mM (**right panel**; 1.5 (dark blue), 1.6 (light blue, dotted), 1.7 (green), 1.8 (yellow, dotted), 1.9 (red), 2.0 mM (gray, dotted)) for 48 h. Each analysis was performed in triplicate, and the experiments were repeated multiple times. (**D**) Antioxidant activity of sulforaphane. Larvae (3.5 dpf) were pretreated with (SF, red) or without ((–), gray, dotted) 40 µM sulforaphane for 12 h, then treated with 2.8 mM H_2_O_2_ (left panel) or 1.9 mM NaAsO_2_ (right panel) for 48 h, and the survival rates were analyzed every 12 h. All survival rates were calculated using the Kaplan–Meier method and the log-rank test was used to compare the variables between the groups; *p* values of < 0.05 were considered to indicate statistical significance.

We have been evaluating the antioxidant activity of Nrf2-activating compounds based on the improvement of the survival rate of zebrafish larvae exposed to toxic oxidative stress [15,27,28]. While the previous method of evaluation required 5 mL of compound solution for each sample (20 larvae in a 35-mm dish), it was better to reduce the volume as much as possible, since many phytochemicals are valuable and expensive. To reduce the volume to one-tenth (0.5 mL), 24-well plates (well diameter, 15.6 mm) were used. First, we determined the appropriate numbers of zebrafish larvae that could be maintained in a healthy condition by performing survival assays in 24-well plates (Figure 1A). Larvae at four days post-fertilization (dpf) were placed in each single well (numbers of larvae, 4, 8, 12, 16 or 20) of a 24-well plate and their status was observed for 120 h (5 days) without exchanging the medium. All larvae survived for 60 h under all conditions, while they became unhealthy after 72 h in a larval-number-dependent manner. We chose eight larvae per well, since they seemed to be healthy, even after 84 h, and set the oxidative stressor treatment time to 48 h (Figure 1B).

Under this condition, we analyzed two oxidative stressors, H_2_O_2_ and NaAsO_2_. We previously showed that 12 h pretreatment with 40 μM sulforaphane was able to reduce the toxicity of further H_2_O_2_ and NaAsO_2_ treatment in zebrafish larvae in a 35-mm dish. To determine the appropriate concentrations of oxidative stressors for a 24-well plate, survival assays were performed using zebrafish larvae (4 dpf). As shown in Figure 1C, the survival rates of H_2_O_2_- or NaAsO_2_-treated larvae were reduced in a dose-dependent manner. We selected 2.8 mM H_2_O_2_ and 1.9 mM NaAsO_2_ as concentrations for further study. Next, we examined the effects of 12 h pretreatment with 40 μM sulforaphane on this assay condition (Figure 1D). The results indicated that sulforaphane significantly reduced the toxicity of these oxidative stressors.

### 2.2. Determination of the Phytochemical Concentration for the Analyses

This zebrafish-based assay system was used to evaluate the antioxidant activity of 10 phytochemicals derived from various spices: capsaicin (chili pepper), carnosic acid (rosemary), cinnamaldehyde (cinnamon), curcumin (turmeric), diallyl trisulfide (garlic), eugenol (clove), 6-gingerol (ginger), isoeugenol (nutmeg), 6-MSITC (wasabi) and quercetin (caper). Since some phytochemicals showed lethal toxicity at a high concentration, the appropriate concentration for the survival assay was determined for each of the 10 phytochemicals. Zebrafish larvae (3.5 dpf) were treated with each of phytochemicals at four different concentrations (1, 5, 25 and 125 µM) for 12 h, and their viability was examined for 48 h. As a result, only larvae treated with eugenol and quercetin survived until 48 h, even at 125 µM (16/16 survivors/tested larvae). For capsaicin, cinnamaldehyde, diallyl trisulfide, 6-gingerol, isoeugenol and 6-MSITC, all larvae survived at 25 µM, but died at 125 µM during the 12-h pretreatment period. With respect to carnosic acid and curcumin, larvae only survived at 1 µM, and at concentrations of >5µM, all larvae died during pretreatment. Based on these results, the antioxidant activities of phytochemicals were not analyzed at lethal concentrations in subsequent experiments.

### 2.3. Protective Effects of Curcumin, Diallyl Trisulfide and Quercetin Against H_2_O_2_ Toxicity

**Figure 2:**
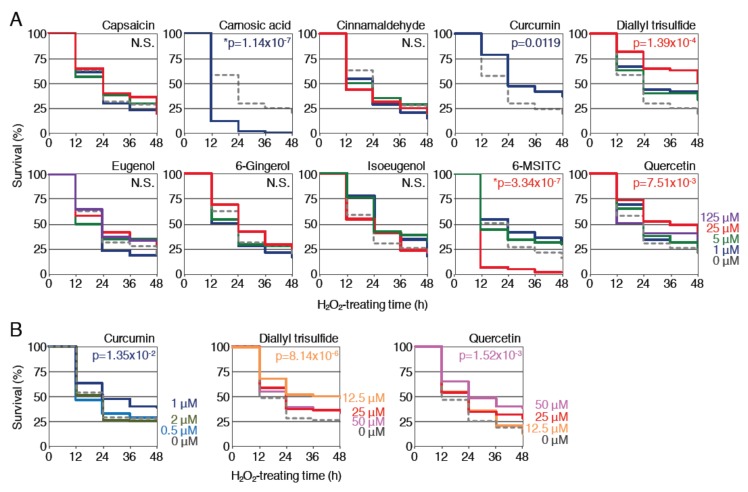
Effects of pretreatment with spice-derived phytochemicals on the survival rates of zebrafish larvae exposed to H_2_O_2_. (**A**) Survival assays using 10 phytochemicals at 4 concentrations. Larvae (3.5 dpf) were pretreated with the indicated phytochemicals at concentrations of 0 µM (gray, dotted), 1 µM (dark blue), 5 µM (green), 25 µM (red) or 125 µM (purple). After pretreatment for 12 h, the solution was changed to 2.8 mM H_2_O_2_ and survival was measured every 12 h for 48 h. Each analysis was performed in triplicate, and the experiments were repeated multiple times. (**B**) Optimization of the concentration of phytochemicals. Larvae (3.5 dpf) were pretreated with curcumin (0 (gray, dotted), 0.5 (light blue), 1 (dark blue), 2 μM (dark green)), diallyl trisulfide (0 (gray, dotted), 12.5 (orange), 25 (red), 50 μM (pink)) or quercetin (0 (gray, dotted), 12.5 (orange), 25 (red), 50 μM (pink)) at the indicated concentrations. All survival rates were calculated using the Kaplan–Meier method and analyzed by log-rank test; *p* values of < 0.05 were considered to indicate statistical significance. Asterisks denote toxic effects of the indicated phytochemicals. N.S. indicates not significant.

First, the effects of pretreatment with each phytochemical against H_2_O_2_ toxicity were examined. Zebrafish larvae (3.5 dpf) were pretreated with each of the phytochemicals at various concentrations for 12 h and then treated with 2.8 mM H_2_O_2_ after the removal of the phytochemicals; the survival rates were analyzed until 48 h and then compared to those without pretreatment (Figure 2A). As a result, the survival of the larvae pretreated with 1 μM curcumin, 25 μM diallyl trisulfide or 25 μM quercetin was significantly increased in comparison to untreated controls. Pretreatment with 5 μM isoeugenol or 5 μM 6-MSITC also tended to increase the survival rate but not to a statistically significant extent (*p* < 0.1). On the other hand, pretreatment with the remaining five phytochemicals was not associated with a clear increase in survival at any concentration. It is noteworthy that the lethality was significantly increased when larvae were pretreated with 1 μM carnosic acid or 25 μM 6-MSITC. For the three positive phytochemicals, we further performed the survival assays against 2.8 mM H_2_O_2_ toxicity to determine the optimal concentration for pretreatment. Zebrafish larvae (3.5 dpf) were pretreated with curcumin (0.5, 1, 2 μM), diallyl trisulfide (12.5, 25, 50 μM) or quercetin (12.5, 25, 50 μM) for 12 h before being treated with 2.8 mM H_2_O_2_ for 48 h, and the survival rates were determined (Figure 2B). As a result, the optimal concentrations for curcumin, diallyl trisulfide and quercetin were determined to be 1, 12.5 and 50 μM, respectively. These results demonstrated that curcumin, diallyl trisulfide and quercetin all exhibited protective effects against H_2_O_2_ toxicity in zebrafish larvae.

### 2.4. Protective Effects of Cinnamaldehyde, Isoeugenol and 6-MSITC against NaAsO_2_ Toxicity

**Figure 3:**
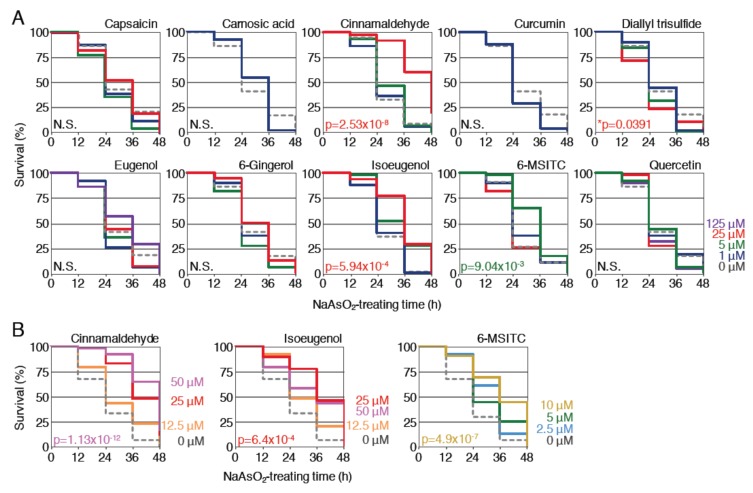
Effects of pretreatment with spice-derived phytochemicals on the survival rates of zebrafish larvae exposed to NaAsO_2_. (**A**) Survival assays using 10 phytochemicals at 4 concentrations. Larvae (3.5 dpf) were pretreated with indicated phytochemicals at concentrations of 0 µM (gray, dotted), 1 µM (dark blue), 5 µM (green), 25 µM (red) or 125 µM (purple). After pretreatment for 12 h, the solution was changed to 1.9 mM NaAsO_2_ and survival was measured every 12 h for 48 h. (**B**) Optimization of the phytochemical concentrations. Larvae (3.5 dpf) were pretreated with cinnamaldehyde (0 (gray, dotted), 12.5 (orange), 25 (red), 50 μM (pink)), isoeugenol (0 (gray, dotted), 12.5 (orange), 25 (red), 50 μM (pink)) or 6-MSITC (0 (gray, dotted), 2.5 (light blue), 5 (green), 10 μM (yellow)) at the indicated concentrations. An asterisk denotes toxic effects of the indicated phytochemical.

The effects of phytochemicals on oxidative stress induced by stressors other than H_2_O_2_ were next investigated. We chose NaAsO_2_, which generates reactive oxygen species (ROS), including superoxide anion, hydroxyl radical and singlet oxygen, in addition to H_2_O_2_ [29]. Larvae (3.5 dpf) were pretreated with each of the phytochemicals at various concentrations for 12 h, and then treated with 1.9 mM NaAsO_2_ after the removal of the phytochemicals; the survival rates were analyzed until 48 h and compared to the survival rates of larvae without the pretreatment (Figure 3A). As a result, the survival rates of larvae pretreated with 25 μM cinnamaldehyde, 25 μM isoeugenol or 5 μM 6-MSITC significantly increased in comparison to untreated controls. Pretreatment with 25 μM capsaicin also tended to increase the survival rate, but not to a statistically significant extent (*p* < 0.1). On the other hand, pretreatment with the remaining six phytochemicals did not increase the survival rates at any concentration. It is noteworthy that the lethality was significantly increased when larvae were pretreated with 25 μM diallyl trisulfide. For the three positive phytochemicals, we further performed survival assays against 1.9 mM NaAsO_2_ toxicity to determine the optimal concentration for pretreatment. Larvae (3.5 dpf) were pretreated with cinnamaldehyde (12.5, 25, 50 μM), isoeugenol (12.5, 25, 50 μM) or 6-MSITC (2.5, 5, 10 μM) for 12 h before being treated with 1.9 mM NaAsO_2_ for 48 h, and the survival rates were determined (Figure 3B). As a result, the optimal concentrations for cinnamaldehyde, isoeugenol and 6-MSITC were 50, 25 and 10 μM, respectively. All of these results demonstrated that cinnamaldehyde, isoeugenol and 6-MSITC exhibited protective effects against NaAsO_2_ toxicity in zebrafish larvae.

### 2.5. Identification of Nrf2-Dependent and Nrf2-Independent Antioxidant Activities of Phytochemicals

To identify whether the antioxidant activities of phytochemicals are Nrf2 dependent, we performed an analysis using Nrf2-mutant zebrafish (*nfe2l2a^fh318^*) [15]. Since Nrf2-homozygous mutant zebrafish were viable and fertile, homozygous-mutant larvae were easily prepared by crossing Nrf2-homozygous adults.

**Figure 4:**
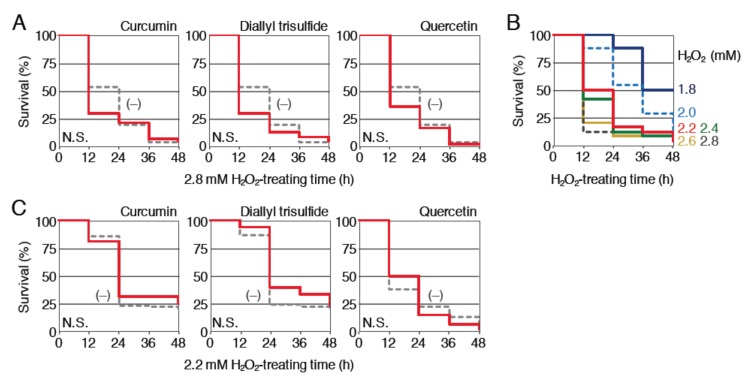
Effects of pretreatment with phytochemicals on the survival rates of Nrf2-homozgous mutant larvae exposed to H_2_O_2_. (**A**) Survival assays using Nrf2-mutant larvae prepared from homozygous mutant parents. Larvae (3.5 dpf) were pretreated with (red; 1 µM curcumin, 12.5 µM diallyl trisulfide or 50 µM quercetin) or without (gray, dotted) indicated phytochemicals. After pretreatment for 12 h, the solution was changed to 2.8 mM H_2_O_2_ and survival was measured every 12 h for 48 h. (**B**) Determination of the optimal H_2_O_2_ concentrations for survival analyses using Nrf2-mutant larvae. Larvae (4 dpf) were exposed to H_2_O_2_ at concentrations of 1.8 to 2.8 mM for 48 h (1.8 (dark blue), 2.0 (light blue, dotted), 2.2 (red), 2.4 (green), 2.6 (yellow), 2.8 mM (dark gray, dotted)). Survival rates were observed every 12 h. (**C**) Survival assays using a lower dose of H_2_O_2_. Nrf2-mutant larvae (3.5 dpf) were pretreated with (red; 1 µM of curcumin, 12.5 µM of diallyl trisulfide or 50 µM of quercetin) or without (gray, dotted) indicated phytochemicals. After pretreatment for 12 h, the solution was changed to 2.2 mM H_2_O_2_ and the survival was measured every 12 h for 48 h.

First, we examined the protective effect of curcumin, diallyl trisulfide and quercetin against H_2_O_2_ toxicity (Figure 4A). Nrf2-homozygous mutant larvae (3.5 dpf) were pretreated with 1 μM curcumin, 12.5 μM diallyl trisulfide or 50 μM quercetin for 12 h and then treated with 2.8 mM H_2_O_2_ after the removal of phytochemicals; the survival rates were analyzed until 48 h and compared with the survival rates of larvae without pretreatment. Pretreatment with the three phytochemicals was not associated with a clear increase in the survival rates, suggesting that the protective effects of these phytochemicals against H_2_O_2_ toxicity were Nrf2 dependent. Since the toxic effects of 2.8 mM H_2_O_2_ were more potent in Nrf2 mutants than in wild-type larvae (compare Figure 2B and Figure 4A), it is possible that Nrf2-independent effects were hidden by this strong lethality. To test this possibility, we next analyzed the effects of three phytochemicals with lower concentrations of H_2_O_2_. Survival assays were performed using Nrf2-homozygous mutant larvae (4 dpf) to determine the appropriate concentration of H_2_O_2_ (Figure 4B). We chose a concentration of 2.2 mM for the treatment of Nrf2 mutants. The effects of 12 h pretreatment with 1 μM curcumin, 12.5 μM diallyl trisulfide or 50 μM quercetin were analyzed using this assay condition (Figure 4C). As a result, none of the three phytochemicals was associated with an obvious improvement in mutant larvae survival, suggesting that their protective effects were indeed Nrf2 dependent.

**Figure 5:**
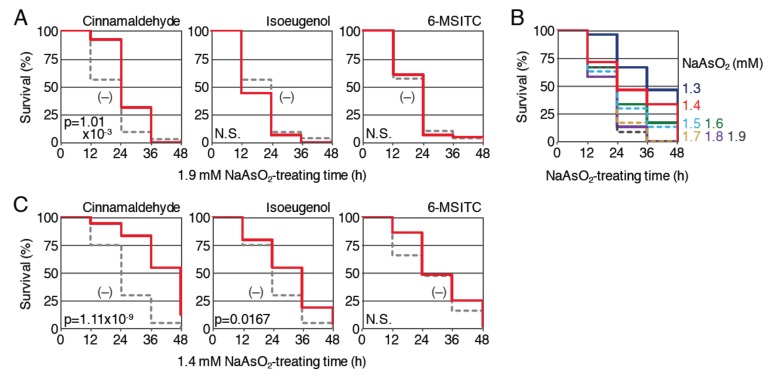
Effects of pretreatment with phytochemicals on the survival rates of Nrf2-homozygous mutant larvae exposed to NaAsO_2_. (**A**) Survival assays using Nrf2-mutant larvae prepared from homozygous mutant parents. Larvae (3.5 dpf) were pretreated with (red; 50 µM cinnamaldehyde, 25 µM isoeugenol or 10 µM 6-MSITC) or without (gray, dotted) indicated phytochemicals. After pretreatment for 12 h, the solution was changed to 1.9 mM NaAsO_2_ and survival was measured every 12 h for 48 h. (**B**) Determination of the optimal NaAsO_2_ concentrations for survival analyses using Nrf2-mutant larvae. Larvae (4 dpf) were exposed to NaAsO_2_ at concentrations of 1.3 to 1.9 mM for 48 h (1.3 (dark blue), 1.4 (red), 1.5 (light blue, dotted), 1.6 (green), 1.7 (yellow, dotted), 1.8 (purple), 1.9 mM (dark gray, dotted)). Survival rates were observed every 12 h. (**C**) Survival assays using a lower dose of NaAsO_2_. Larvae (3.5 dpf) of Nrf2 mutant were pretreated with (red; 50 µM cinnamaldehyde, 25 µM isoeugenol or 10 µM 6-MSITC) or without (gray, dotted) indicated phytochemicals. After pretreatment for 12 h, the solution was changed to 1.4 mM NaAsO_2_ and survival was measured every 12 h for 48 h.

Second, we examined the protective effect of cinnamaldehyde, isoeugenol and 6-MSITC against NaAsO_2_ toxicity (Figure 5A). Nrf2-homozygous mutant larvae (3.5 dpf) were pretreated with 50 μM cinnamaldehyde, 25 μM isoeugenol or 10 μM 6-MSITC for 12 h and then treated with 1.9 mM NaAsO_2_ after the removal of the phytochemicals; the survival rates were analyzed until 48 h and compared with the survival rates of larvae without pretreatment. Pretreatment with isoeugenol and 6-MSITC was not associated with a clear increase in the survival rate, suggesting that the protective effects of these phytochemicals against NaAsO_2_ toxicity were Nrf2 dependent. However, the survival rates of the larvae pretreated with cinnamaldehyde were significantly increased in comparison to untreated controls, indicating that the anti-NaAsO_2_ activity of cinnamaldehyde is, at least partially, Nrf2 independent. As was the case with H_2_O_2_, the Nrf2-independent antioxidant effect of each phytochemical may have been hidden by the potent toxicity of 1.9 mM NaAsO_2_ in Nrf2-mutant larvae; thus, we analyzed the effects of three phytochemicals with a lower concentration of NaAsO_2_. Survival assays were performed using Nrf2-mutant larvae (4 dpf) to determine the appropriate concentration of NaAsO_2_, and we selected 1.4 mM for the further experiments using Nrf2 mutants (Figure 5B). The effects of 12 h pretreatment with 50 μM cinnamaldehyde, 25 μM isoeugenol or 10 μM 6-MSITC were analyzed using this assay condition (Figure 5C). As a result, larvae pretreated with 6-MSITC showed no obvious difference from untreated larvae, while pretreatment with the other two phytochemicals was associated with a significant improvement in the survival of the Nrf2-homozygous mutants. These results suggest that the anti-NaAsO_2_ activities of 6-MSITC, isoeugenol and cinnamaldehyde are mainly dependent on Nrf2, Nrf2 plus other pathways and only other pathways, respectively.

## 3. Discussion

**Figure 6:**
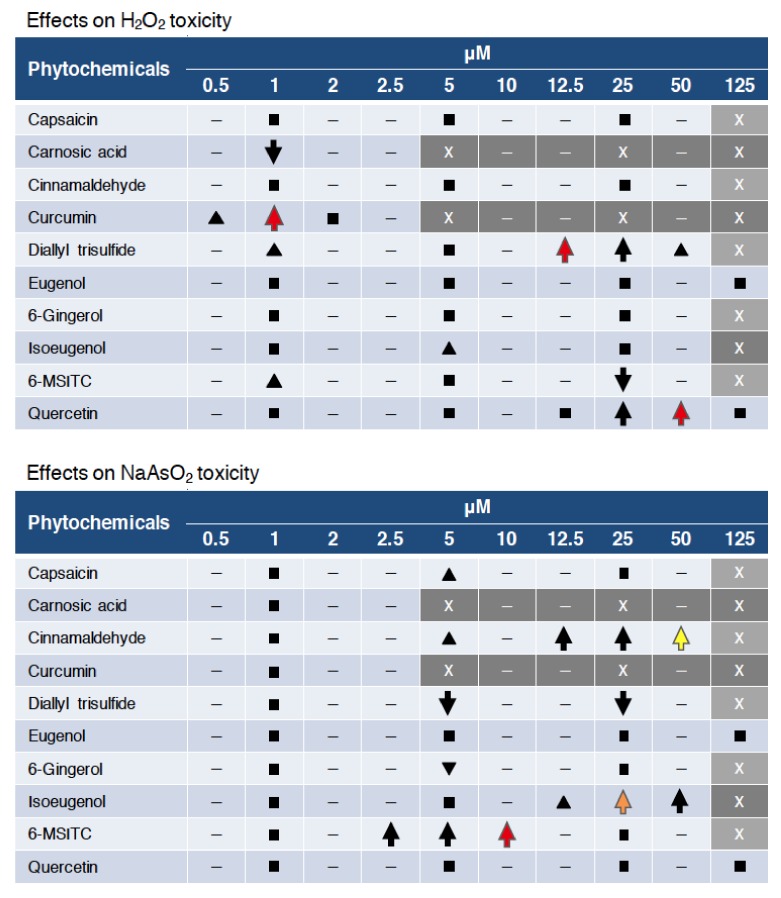
Summary of the effects of phytochemical pretreatment. Up and down arrows denote the survival effects, with a significant increase or decrease, respectively, in survival (*p* < 0.05). Red, orange and yellow indicate Nrf2-depedent, partially Nrf2-depedent and Nrf2-independent activities, respectively. Black triangles denote a non-significant increase (*p* < 0.1). Squares mean no significant effects. “–” denotes “not analyzed”. “X” and gray background indicate toxic concentrations for each phytochemical.

In this study, we established an assay system to evaluate the antioxidant activity of phytochemicals using zebrafish and utilized this system to compare the protective activities of 10 spice-derived phytochemicals against two oxidative stressors, H_2_O_2_ and NaAsO_2_. As a result, curcumin, diallyl trisulfide and quercetin showed preventive activities against H_2_O_2_ toxicity, while cinnamaldehyde, isoeugenol and 6-MSITC displayed preventive activities against NaAsO_2_ toxicity (Figure 6, up arrows). Furthermore, most of these activities were reduced in Nrf2-mutant zebrafish, suggesting that the Nrf2 pathway was the major biological target of these antioxidant phytochemicals (Figure 6, red & orange). Interestingly, the protective effect of cinnamaldehyde against NaAsO_2_ toxicity was Nrf2 independent, indicating the existence of antioxidant mechanisms other than the Nrf2 pathway (Figure 6, yellow). These unique findings have been observed for the first time, probably because of the use of zebrafish in the phytochemical evaluation. We therefore believe that it would be worthwhile to conduct a comprehensive analysis of various other phytochemicals using the zebrafish-based assay system in the future.

Since all phytochemicals used in this study were known to have antioxidant activity [30,31,32,33,34,35,36,37,38,39], we considered that almost all of the 10 phytochemicals would also exhibit antioxidant activity in zebrafish larvae. However, only five showed protective effects against H_2_O_2_ toxicity, including isoeugenol and 6-MSITC, which showed only slight effects (Figure 6, black triangles), in addition to curcumin, diallyl trisulfide and quercetin. As for NaAsO_2_ toxicity, four phytochemicals (cinnamaldehyde, isoeugenol, 6-MSITC and capsaicin) showed protective effects, with capsaicin showing only a slight effect (Figure 6, black triangles). Carnosic acid, eugenol and 6-gingerol did not show any of protective effects against either oxidative stressor. Interestingly, the phytochemicals that were effective against H_2_O_2_ toxicity differed from those that were effective against NaAsO_2_ toxicity. It is known that arsenite produces not only H_2_O_2_ but also other ROS, such as superoxide anions, via the activation of NADPH oxidase [29] and also inhibits antioxidant proteins through binding to their cysteine residues [40]. We hypothesized that the different effects of each phytochemical may be based on the different toxic mechanisms of the two oxidative stressors. It would be interesting to analyze other types of oxidative stressors in the future.

The most interesting finding was that cinnamaldehyde demonstrated a greater protective effect against NaAsO_2_ toxicity than sulforaphane in an Nrf2-independent manner (see Figure 1D, Figure 3A and Figure 5A,C). The effects of cinnamaldehyde on NaAsO_2_ toxicity were also shown by Wondrak et al. [41] using HCT116 colon cancer cells. However, they considered that this effect was due to the activation of the Nrf2 pathway. Cinnamaldehyde is known to target biological pathways other than the Nrf2 pathway, including TRPA1, a member of the transient receptor potential (TRP) cation channel family [42,43]. We would like to clarify which pathways contribute to this antioxidant activity in the future. An Nrf2-independent effect against NaAsO_2_ toxicity was also observed in the case of isoeugenol. The effects of isoeugenol are interesting because of following reasons: (1) it exhibited both Nrf2-dependent and Nrf2-independent activities against NaAsO_2_ toxicity; (2) it displayed some protective effects against H_2_O_2_ toxicity; and (3) it has been reported to have stronger antioxidant and Nrf2-activating activities than its analogous compound, eugenol [37,44]. The molecular mechanism underlying isoeugenol-mediated cytoprotection is worth clarifying.

Diallyl trisulfide, 6-MSITC and quercetin all showed Nrf2-dependent antioxidant activity. Diallyl trisulfide showed protective effects against H_2_O_2_ toxicity at concentrations of 12.5 and 25 μM, but against NaAsO_2_ toxicity it induced lethality, even at 5 μM, suggesting the cotoxicity of diallyl trisulfide and arsenite. Previous reports showed that diallyl trisulfide not only ameliorates H_2_O_2_ toxicity in C2C12 skeletal muscle myoblast cells in a Nrf2-dependent manner [45], but also protects the rat liver from arsenic acid damage [46]. It is not known whether this discrepancy is due to the difference in arsenic forms, tissues/cells or species. In contrast to the findings with diallyl trisulfide, pretreatment with 25 μM 6-MSITC showed Nrf2-dependent protection against NaAsO_2_ toxicity, while it exhibited cotoxicity in the case of H_2_O_2_ toxicity. 6-MSITC is an isothiocyanate similar to sulforaphane and has been shown to protect against NaAsO_2_ toxicity in mouse primary hepatocytes [47] and to induce phase 2 detoxifying genes in the mouse liver in an Nrf2-dependent manner [48]. It is plausible that the zebrafish larvae also exhibited antioxidant activity through the Nrf2 pathway. However, 6-MSITC has also been reported to reduce the toxicity of H_2_O_2_ in rat fetal striatal cells [38], which was not observed in our case, suggesting that the regulation may differ between tissues/cells or species. Quercetin showed a protective effect against H_2_O_2_ toxicity at concentration of 25 and 50 μM in an Nrf2-dependent manner but had no effect on NaAsO_2_ toxicity. Quercetin is one of the most well-known Nrf2-activating compounds used in this study [49] and has been reported to be effective against damage caused by long-term NaAsO_2_ treatment in the rat liver and brain [50]. The discrepancy between the latter report and our current study may be due to acute toxicity caused by the high concentration of NaAsO_2_. However, quercetin seems to be a highly safe phytochemical, at least in zebrafish larvae, as it did not kill the larvae even when used at a high concentration of 125 μM.

Four phytochemicals did not show significant protective effects against the two oxidative stressors. Among these, capsaicin has various properties, including pain relief, weight loss, body thermoregulation, antimicrobial and anticancer activities, probably through its receptor TRPV1, another member of the TRP family [51]. Although there are many reports on the antioxidant effects of capsaicin, the molecular basis of its action, including its relationship to the Nrf2 pathway, has not been well understood [30]. It is likely that capsaicin indirectly activates some cellular antioxidant mechanisms, but it may not function well in zebrafish larvae. Carnosic acid has been reported to exhibit antioxidant activity through the Nrf2 pathway in various cells and mice [31,52]. However, in zebrafish larvae, the toxicity of carnosic acid was so strong that we could not analyze it at concentrations ≥5 μM, and even at 1 μM, the numbers of surviving larvae were significantly decreased due to cotoxicity when they were further treated with H_2_O_2_. We consider that the toxicity will emerge before its medicinal effects appear. Eugenol has been reported to show antioxidant activity [35]; however, the relationship between its activity and the Nrf2 pathway was not clear. In zebrafish larvae, eugenol did not show any antioxidant activity or toxic effects, even at 125 μM. In the case of 6-gingerol, the antioxidant activity is less well known than the anti-inflammatory effect [36]. We consider that the antioxidant activity of 6-gingerol may be too weak to enhance the survival of zebrafish larvae.

In this study, we used zebrafish larvae to analyze the antioxidant activity of phytochemicals derived from food ingredients and their Nrf2 dependency. This was the first study to simultaneously evaluate the antioxidant activity of 10 or more phytochemicals in a vertebrate body. We used more than 48 larvae per single measured value, which might be difficult to do using mice or other mammals due to economic reasons and ethical considerations. It would also be difficult to treat mice uniformly with these phytochemicals. Because of the small size of zebrafish larvae (length 2 mm) and the fact that they are aquatic, they can be uniformly treated with drugs, similarly to cultured cells. The 5 mL of compound solution required in our previous assay system using a 35-mm dish [15] might be too much for the analysis of valuable and expensive phytochemicals. In the improved assay system described in the present study, a 24-well plate was used to reduce the amount of the phytochemical solution to 0.5 mL per well, which allowed us to reduce the amounts of rare samples that were used. Only 10 spice-derived phytochemicals were analyzed in this pilot study, nevertheless, we could find both oxidative-stressor-specific effects and Nrf2-independent activity, suggesting that the zebrafish-based assay system will be useful for evaluating the properties of phytochemicals. The zebrafish-based assay system is relatively low-throughput in comparison to systems using cultured cells, but has several advantages: (1) it reflects effects on multicellular tissues in physiological conditions; (2) it uses normal, but not cancer, cells; (3) developmental toxicity and teratogenicity can be evaluated at the same time; and (4) the molecular mechanism can be genetically identified using gene knockout lines. It is therefore considered to be useful for the comprehensive evaluation of various food ingredients and for screening unknown beneficial phytochemicals from food extracts. Although the zebrafish is a fish, we consider that it is possible to apply the results obtained from zebrafish-based system to human health by verifying them using a mammalian model. In the future, we will use this assay system to screen and evaluate antioxidant activities in various foods, such as vegetables, fruits, beans and dairy products.

## 4. Materials and Methods

### 4.1. Zebrafish and Chemicals

In this study, wild-type (AB strain) and Nrf2-mutant (*nfe2l2a*^*fh318*^) [15] zebrafish larvae were used. The Nrf2-mutant line was maintained by polymerase chain reaction based genotyping, as described previously [28]. Embryos were obtained by natural mating. Capsaicin, carnosic acid, cinnamaldehyde, eugenol, 6-gingerol, isoeugenol, quercetin, H_2_O_2_ and NaAsO_2_ were purchased from FUJIFILM Wako (Osaka, Japan). Diallyl trisulfide and sulforaphane were bought from LKT Laboratories (St. Paul, MN, USA). Curcumin and 6-MSITC were purchased from Sigma-Aldrich Japan (Tokyo, Japan) and Abcam (Cambridge, UK), respectively. H_2_O_2_ and NaAsO_2_ were dissolved in MiliQ water (Merck-Millipore, Billerica, MA), sulforaphane in ethanol and other phytochemicals were dissolved in dimethyl sulfoxide for the stock solution, and were diluted to the final concentration with E3+ medium (5 mM NaCl, 0.17 mM KCl, 0.33 mM CaCl_2_, 0.33 mM MgSO_4_ and 0.1 μg/mL methylene blue).

### 4.2. Survival Assays

Survival assays were performed as previously described [28] with slight modification. Briefly, larvae (3.5 dpf, 8 larvae per well in a standard condition) were placed in each well of a 24-well plate, instead of a 35-mm dish in the previous report, with 500 µL of phytochemical solution (E3+ medium containing each phytochemical) for 12 h. At 4 dpf, the phytochemical solution was replaced with oxidative stressor solution (E3+ medium containing H_2_O_2_ or NaAsO_2_). We selected 2.8 mM H_2_O_2_ and 1.9 mM NaAsO_2_ as optimized concentrations to evaluate the antioxidant activity of phytochemicals. The survival of larvae was observed for 48 h after starting the oxidative stressor treatment. Each analysis was performed in triplicate, and the experiments were repeated multiple times to confirm reproducibility. Larvae were not fed during the experiment. All animal experiments were performed in accordance with the animal protocols approved by the Animal Experiment Committee of the University of Tsukuba (Approval Identification Number: 18368; approval date: 1st June 2018). All methods were carried out in accordance with the Regulation for Animal Experiments in our University and the Fundamental Guideline for Proper Conduct of Animal Experiment and Related Activities in Academic Research Institutions under the jurisdiction of the Ministry of Education, Culture, Sports, Science and Technology.

### 4.3. Statistical Analysis

All survival data were calculated using the Kaplan–Meier method and analyzed by log-rank test; *p* values <0.05 were considered to indicate statistical significance. All statistical analyses were performed using EZR [53], which is a graphical user interface for R (The R Foundation for Statistical Computing, Vienna, Austria). More precisely, it is a modified version of R commander designed to add statistical functions frequently used in biostatistics.

## Abbreviations

dpf	days post-fertilization
H_2_O_2_	hydrogen peroxide
6-MSITC	6-(methylsulfinyl)hexyl isothiocyanate
NaAsO_2_	sodium arsenite
Nrf2	NF-E2-related factor 2
TRP	transient receptor potential
